# Practice Nurses' views of their role in the management of Chronic Fatigue Syndrome/Myalagic Encephalitis: a qualitative study

**DOI:** 10.1186/1472-6955-8-2

**Published:** 2009-01-22

**Authors:** Carolyn Chew-Graham, Rebecca Dixon, Jonathan W Shaw, Nina Smyth, Karina Lovell, Sarah Peters

**Affiliations:** 1 School of Community-Based Medicine, University of Manchester, Manchester, M13 9PL, UK; 2 School of Psychological Sciences, University of Manchester, Manchester, M13 9PL, UK; 3 School of Nursing, Midwifery and Social Work University of Manchester, Manchester, M13 9PL, UK

## Abstract

**Background:**

NICE guidelines suggest that patients with Chronic Fatigue Syndrome/Myalgic Encephalitis (CFS/ME) should be managed in Primary Care. Practice Nurses are increasingly being involved in the management of long-term conditions, so are likely to also have a growing role in managing CFS/ME. However their attitudes to, and experiences of patients with CFS/ME and its management must be explored to understand what barriers may exist in developing their role for this group of patients. The aim of this study was to explore Practice Nurses' understanding and beliefs about CFS/ME and its management.

**Methods:**

Semi-structured interviews with 29 Practice Nurses. Interviews were transcribed verbatim and an iterative approach used to develop themes from the dataset.

**Results:**

Practice nurses had limited understanding about CFS/ME which had been largely gained through contact with patients, friends, personal experiences and the media rather than formal training. They had difficulty seeing CFS/ME as a long term condition. They did identify a potential role they could have in management of CFS/ME but devalued their own skills in psychological intervention, and suggested counselling would be an appropriate therapeutic option. They recognised a need for further training and on going supervision from both medical and psychological colleagues. Some viewed the condition as contentious and held pejorative views about CFS/ME. Such scepticism and negative attitudes will be a significant barrier to the management of patients with CFS/ME in primary care.

**Conclusion:**

The current role of Practice Nurses in the ongoing management of patients with CFS/ME is limited. Practice Nurses have little understanding of the evidence-base for treatment of CFS/ME, particularly psychological therapies, describing management options in terms of advice giving, self-help or counselling. Practice Nurses largely welcomed the potential development of their role in this area, but identified barriers and training needs which must be addressed to enable them to feel confident managing of patients with this condition. Training must begin by addressing negative attitudes to patients with CFS/ME.

## Background

Chronic Fatigue Syndrome (CFS) or Myalgic Encephalitis (ME) is a symptomatically defined condition with a principal complaint of severe, persistent, disabling fatigue which has been present for 6 months or more [1]. The symptom of fatigue must be of new or definite onset (i.e. not lifelong), and produces a substantial reduction in the patient's previous level of occupational, educational, social, or personal activities [2]. The diagnosis of CFS/ME remains controversial and debated but it is increasingly recognised as a clinical entity in primary care, although only about half of general practitioners/family physicians (GPs/FPs) feel confident with making the diagnosis [3].

A recent report produced for the UK Chief Medical Officer by the CFS/ME working group, placed UK population prevalence at least 0.2–0.4% [4], being twice as common in women as in men, and that the syndrome affects all social classes and ethnic groups to a similar extent. Wessely et al. found the point prevalence of CFS/ME to be 2.6% [5], and that rates did not vary with social class.

The Chief Medical Officer report stated that patients should be diagnosed earlier and given better access to treatment, with a mutual management approach as its key component. This approach should be patient-centred therapy delivered by a trained multi-disciplinary team. The report also emphasised the need to provide these services locally [4]. This approach is echoed in the NICE guidelines for CFS/ME [6] which advocates a prominent role for primary care and that healthcare professionals should aim to establish a supportive and collaborative relationship, working in partnership with the adult or child with CFS/ME, family, and carers to facilitate their effective management.

Practice nurses provide a substantial amount of care to patients in UK primary care. Practice nurses are registered general nurses who have varied post registration qualifications and expertise. They are employed by the GP/FP. Their role is being increased in the management of patients with long term conditions [7] but currently have little or no formal input into the care of patients with CFS/ME. It has been proposed that nurses might have a potential role in the management of patients with CFS/ME due to their ability to provide holistic care [8], developing a relationship and validating the patient's illness experience, identifying patients' expressed needs, and providing appropriate information [8]. A potential role of nurses in the management of patients with medically unexplained symptoms (MUS) [9] has been suggested as a care co-ordinator who gets to know and support not only the patient, but also *cares*. Such a role could be especially useful for the more severely affected CFS/ME patients who experience barriers in accessing all forms of health care. It has been suggested that these patients could be managed by nurses with programmes structured around mobility, personal care and communication [10].

The expansion of the nursing role in the management of patients with CFS/ME is, however, not without barriers. Attitudes of practice nurses to CFS/ME are unknown, but there is evidence amongst GPs/FPs of scepticism towards CFS/ME patients [11,12] who, unsurprisingly therefore, are generally dissatisfied with the medical care they receive [13]. Many doctors perceive CFS/ME patients to have undesirable traits, and it has been argued that these attitudes towards patients often lead to problems with management [11]. The origin of these attitudes were perceived to be a lack of precise bodily location for the complaints, and the reclassification of the syndrome over time, producing conflict between doctor and patient over causation and management [14] and an apparent unwillingness of patients to disclose psychosocial components of their illness models [15]. Previous literature provides evidence of doctor-patient conflict due to disagreement over the causes and management of CFS [12], and of more positive attitudes amongst GPs/FPs who accept CFS/ME as a recognizable clinical entity [16].

Barriers have been described which might reduce the potential for involving practice nurses in the management of patients with emotional problems that are often associated with a chronic illness: there is evidence that practice nurses are not, and do not feel, fully prepared to work with patients with conditions such as depression, and may be unmotivated to acquire the necessary further skills [17]. A comparative study examining practice nurses' current experiences of caring for patients with mental health problems found 52% lacked any formal mental health training [18] but the majority were keen to expand their role and knowledge in mental health care if appropriate support and training was offered to cover the training needs they identified. There are no previous studies which explore practice nurses' attitudes to patients with CFS/ME in primary care.

The NICE guidelines encourage practice nurses to take an active role in the management of CFS/ME in primary care, and policy dictates that nurses should play a bigger role in managing patients with long term conditions, it is thus essential to explore their beliefs about CFS/ME and attitudes towards patients suffering with this condition. It is also important to understand practice nurses' views on what role they might play in management of patients with CFS/ME and identify any training needs. This was the aim of the study reported here.

## Methods

This qualitative study had been reviewed and approved by the Eastern MREC (reference 03/5/62) and had PCT (Primary Care Trust) R&D approval. Semi-structured interviews were carried out with practice nurses in North West England working in practices that had been previously approached to participate in the FINE trial [19]. Sampling for this qualitative study was purposive and sought to achieve maximum variation in relation to nurses': age, practice location and size of practice and length of time as a practice nurse. This was not a representative sample of practice nurses, rather a purposive sample in order to access a range of views. Seventy practice nurses in participating practice were invited by letter with an accompanying information sheet and contacted subsequently by telephone to discuss the study and ask them to participate in an interview. Of these, 29 agreed to be interviewed. Many of those who declined stated when contacted by telephone that they would not be interviewed because they knew very little about CFS/ME. Semi-structured interviews were conducted (by RD, JS and NS) between June 2006 and February 2008. Interviews lasted between 12 and 45 minutes. All participants were female and were registered general nurses, but with a variety of additional qualifications. The mean length of time as a practice nurse was 9.3 years (range 1–23). Further participant details are presented in Table 1.

**Table 1:** Details of study participants

ID	Years as Practice Nurse	Number of Practice Nurses (including respondent) in the practice	Practice location*	Practice list size (thousands)
1	12	3	IC	12
2	6	2	IC	6.5
3	20	1	S	3.5
4	3	1	S	3.2
5	1.5	3	R	8.4
6	1	3	R	9
7	12	1	R	2.5
8	13	3	IC	7.7
9	16	3	IC	7.7
10	10	3	IC	8.2
11	9	2	IC	6
12	2	3	S	9
13	3	4	S	16
14	1	4	S	16
15	2	1	R	2.9
16	4	2	IC	5.6
17	9	1	IC	2.4
18	12	3	S	8.6
19	7	4	IC	12
20	4.5	2	S	4.5
21	19	4	S	6.7
22	3.5	4	S	6.7
23	8	4	IC	1.2
24	7.5	4	IC	1.2
25	11	5	IC	16
26	23	2	IC	5.8
27	23	3	S	6.7
28	21	5	IC	8

29	6	2	S	6.7

* IC = inner city; S = suburban; R = rural

An interview guide provided a flexible framework for questioning and explored a number of areas: the cause of CFS/ME, previous experience of patients with CFS/ME, potential role and anticipated training needs, and experience of the psychological management of patients with long term conditions. The interviewer combined open questions to elicit free responses with focused questions for probing and prompting. Interviews were digitally recorded and transcribed verbatim.

Analysis proceeded in parallel with the interviews and was inductive, taking an interpretative stance [20,21]. Transcripts were read and discussed by researchers from different professional backgrounds (primary care, psychology and nursing) so increasing the trustworthiness of the analysis [22]. Coding was iterative and was informed by the accumulating data and continuing thematic analysis. Thematic categories were identified in initial interviews which were then tested or explored in subsequent interviews where disconfirmatory evidence was sought [20]. Interpretation and coding of data was undertaken by CCG, SP, KL, RD, JS and NS individually and the themes were agreed through discussion. The importance of reflexivity [20] was discussed within the research team, with medical researchers (CCG, JS and RD) particularly reflecting on how their clinical perspective impacted on collection, analysis and interpretation of the data. In reporting the final analysis the data are presented to illustrate the range and commonality of meaning of each category.

## Results

Data are presented verbatim and the practice nurse identifier is displayed in brackets. Data are organised into six themes: i) Developing the knowledge; ii) Understanding the condition; iii) Understanding the patient; iv) Labelling and diagnostic uncertainty; v) Using existing skills; vi) Developing another role.

### i) Developing the knowledge

Without exception, practice nurses reported having limited experience of working with patients with CFS/ME and no (or extremely limited) training and training opportunities. Indeed several practice nurses approached to take the part in the study were initially reluctant stating they didn't feel they had anything to offer on the subject:

*'I mean we all sort of know it's there but, but no, well I, I personally think I've never, had any formal training and never, heard of any, particularly.' *(PN11)

As a consequence, they were uncertain about how the condition presented:

*'I'll be honest and say that i'm a little bit ignorant to it ermm...symptoms: lethargy, poor concentration, maybe some symptoms of depression with it...not working, no motivation.' *(PN10)

All were aware of having a knowledge gap in this topic:

*'I know so little you could write it on a postage stamp what I know.' *(PN21)

They reported that any previous training had been coloured by personal opinions that senior staff held, with patients with CFS/ME being portrayed in a derogatory manner:

*'Early on in my training it was referred to as ME even though it isn't now. Erm, but it was seen as the malingerers last resort really for people who wanted to be more interesting than anyone else but, wanted not to do very much with their life. Erm*, [pause], *great deal of scepticism and, no time paid to it whatsoever, brushed over.' *(PN16)

Rather than formal training, their knowledge had been gleaned from media coverage.

*'The only person I know that had CFS is... Esther Rantzen's daughter...had this for years, she was often on the telly talking about it...perhaps if she hadn't had it I wouldn't even know about it.' *(PN25)

In addition, an important source of knowledge came from encounters with personal friends who had the condition. Largely this led them to become more accepting of it as a clinical problem and shaped their views on aetiology:

*'My view has been changed since my friend got it...I used to think it was people who were more stressed who would be more prone to suffer from it. That they were bordering on having a nervous breakdown, depression and these other symptoms that they didn't know. But since then I don't, I think it is a disease*.' (PN3)

More powerful still in developing their views was personal experience of the condition. This allowed practice nurses to identify with patients more easily, and to empathise with the symptoms they were describing:

*'I suppose I differ in the way that I had a virus, a viral illness many years ago and it was so bad I could barely lift my head, and no-one knew the cause of it, all my blood pattern was normal, I just could not get up... I didn't know what was happening to me and I, I did feel, in the end, is this some form of depression? You know, is it medical? Is it mental? I just did not know. All I know is how I felt, absolutely dreadful. And it didn't matter what the cause for that was to me, it was how I was and that's all that matters, so, I do try to look at it that way.' *(PN19)

### ii) Understanding the condition

Given the limited formal education and training, it was unsurprising that practice nurses held a wide range of illness models of CFS/ME. These informed their views about how the condition should be managed. Whilst some viewed CFS/ME as having its origins in a physical disease process (usually a viral infection or compromised immunity system) most considered it as a multifaceted condition and described CFS/ME patients using a biopsychosocial model:

*'Well the whole things are mixed up. I mean presumably it must have some physical basis if its, post-viral *[pause], *but if you're battling on and on and on trying to get people to believe you feel dreadful then it must be very debilitating emotionally as well. So it's chicken and eggs, isn't it?' (PN13)*

Although practice nurses often recognized the disorder had many components, some presented conflicting beliefs within a single narrative, revealing an incoherent illness model:

*'They have a change in their white count levels, they've usually had a virus, a recent viral infection that can trigger it...depression, I think it's all related to depression...all were middle class women, erm, between the ages 30 and 50...I don't think it's a real condition.' *(PN26)

Such narratives use mind-body dualism, without being able to integrate the two approaches.

Other nurses saw the problem as being caused by features of modern life:

*'because of modern appliances the actual physical side of life is easier...there's a lot more stress in today's society and we're all much much busier...that takes it's toll and you do feel more exhausted because of that erm and also in you know like 60 years ago people had the support of their family which for most societies today that's not there.' *(PN28)

Or how individual personalities responded to their environment:

*'It's the copers and the non-copers...some people will cope with life and whatever you throw at them they will get on with it and some people can't cope.' *(PN26)

Some practice nurses questioned the legitimacy of CFS/ME patients' symptoms:

*'I mean there are a few who spoil it for those who are "genuine", they come in and have all these symptoms that don't match up and you just know in the next few months the DLA forms will be coming through.' (*PN4)

### iii) Understanding the patient

In general, participants were positive about patients they had encountered, recognising CFS/ME as a physically and psychologically impairing condition:

*'Anyone who has genuinely got CFS I think it's very sad because they can't live a normal life, you know? It precludes them from everything, enjoying their normal day to day things that we take for granted.' *(PN24)

However, some nurses revealed attitudes to CFS/ME that were less understanding than those they held towards other long term conditions:

*'I see the ones with rheumatoid arthritis...they come in and sit down and say 'oh I'll be alright'. They get on with it. Then, you know, you got these people chronic fatigue, and you think 'what's all that about?' You wanna shake people sometimes.' *(PN26)

This was not exclusive to physical disorders:

*'I do have some sympathy with a real clinical depression, but chronic fatigue...it's such a new thing isn't it?' *(PN26)

Those who were sceptical of the genuineness of the condition also held negative attitudes suggestion patient duplicity:

*'I would suspect...lay people...would think people just don't want to work – they're lazy...if you're working with people who is permanently...off sick, I sure think people would think 'oh gosh, flipping, they are off again'. I don't know, I think there are certainly negative views.' *(PN25)

A number of practice nurses used explicitly negative and pejorative terms to describe patients with CFS/ME:

*'If all those come back normal [investigations] well, I don't know what they call them. They probably call them lazy fags... they probably classed as a waste of space... because [pause] people probably just look at them and think, oh you know he's just tired all the time: lazy...lazy bastards and wasting doctor's time.' *(PN29)

### iv) Labelling and diagnostic uncertainty

Generally, participants felt that reaching a diagnosis was useful for patients and for the service:

*'It's a relief for a lot of patient when they actually informed that they've got it...its relief when they have been given diagnosis.' *(PN24)

Reaching a diagnosis was believed to be problematic because there is no definitive diagnostic test available to confirm CFS/ME:

*'But as you know there's no test for chronic fatigue syndrome, there's you know, you cannot, take some blood and say "YESSS we've got chronic fatigue syndrome", cos there's nothing, you know, its fuzzy.' *(PN10)

Providing a label was seen as the responsibility of the GP/FP and this meant that the views of the doctors in the practice of the condition were critical:

*'I think it is very much related to GPs, some GPs are sympathetic to it and some in my last practice – there were four doctors, and two were sympathetic and two weren't at all. So they would never, never diagnose chronic fatigue.' *(PN26)

The contentious nature of CFS/ME as a recognised clinical syndrome was actively discussed. The views expressed echoed the published literature showing that the diagnosis is viewed with some scepticism among many health professionals:

*'you know what general like people are like, they're like skivers, err, wh-who pretend that they have all these things because you can get a lot of stuff on the internet now, so they present themselves with lots of these symptoms and you think ah maybe that person's got ME or, or, or something similar.' *(PN13)

Other practice nurses saw labels as problematic and potentially stigmatising, and considered the importance of keeping an open mind, instead focusing on and managing the suffering, regardless of the cause or label:

*'everyone's an individual you can't just label...it's very easy to label a patient as being a nuisance or a malinger or an hypochondriac when in actual fact this patient presenting with these symptoms and feeling unwell and is affecting her life you know her quality of life is affected.' *(PN24)

### v) Using existing skills

Regardless of their views about the aetiology of the condition, practice nurses felt that CFS/ME is a difficult condition to manage:

*'I could imagine it could be quite time consuming and possibly frustrating at times, 'cos it's a, like one step forward and two back.' *(PN13)

The only role nurses reported they currently played in the management of patients with CFS/ME was to conduct investigations delegated by the GP/FP to exclude physical causes of the patients' symptoms:

*'As a general practice nurse I'd probably have to clinically rule out any physical reasons for it, have a full screen of bloods, because there are many illnesses which givs symptoms the same, so we would have to sort of work through the physical.' *(PN 22)

However, they did identify a number of ways they worked with other patients which was directly relevant to working with CFS/ME patients. They considered that they took a more empathic approach to managing patients than other health professionals and that this would be important for this group of patients:

'*Patients say the nurse is more approachable, the nurse seems to be listening, they sometimes feel the doctor is not listening to what they are saying because they are limited to a certain amount of time per consultation.' *(PN4)

Because of this relationship, nurses reported that patients confided in them and shared information:

*'From what patients say on a daily basis they find it easier to talk to a nurse than they do to a GP...there's still this feeling that the GP is better, erm, whereas the nurse they find they can be more open...the nurse isn't going to judge them.' *(PN28)

As well as describing themselves as having good listening skills and experience in facilitating patients' disclosure, they also described other ways in which they currently worked psychologically with patients with long term conditions. Primarily they described this in terms of education and motivation around lifestyle issues:

*'A lot of motivation and encouragement and just sort of reinforcement of information they already know.' *(PN25)

*'Making sure they have a healthy diet, hopefully making sure they are taking a variety of foods...small amounts of foods, one would hope they wouldn't be eating copious amounts of all the wrong things, high calories, because if they are not getting out and doing anything they are going to pile the weight on, which is going to make things worse for them anyway its going to make their tiredness even worse isn't it?' *(PN21)

They could see how this could be directly applicable to CFS/ME patients:

*'You know it's been proven that we're good motivators, so, I think we're probably, there is a role for us.' *(PN14)

*'Anybody that has any, erm, erm disease, or, erm symptom that is worrying them, if you can talk it through with them, and this isn't just chronic fatigue its for anything, it, you can make a difference, you know, by helping support, giving information, correct information, correcting any, sort of, old wives tales.' *(PN17)

They also described developing tailored goals to patients' abilities and needs, similar to techniques used in graded exercise therapy:

*'Assessing what they can and cannot do...exercise within their limits because they would probably get more tired than anyone else'*. (PN24)

However, they were not confident about their current expertise and eligibility in using more formal psychological interventions and were reluctant to label what they did in this way.

*'I suppose we do that CBT, in a very sort of unqualified way...I suppose it does come, slip in somewhere.' *(PN27)

Without exception the practice nurses interviewed did not feel they were sufficiently skilled to intervene psychologically with CFS/ME patients:

*'I wouldn't be prepared to be involved without actually having some training, so if they want me to do it, I don't have a problem...I want to be trained in it...I don't want to fly off the seat saying things I haven't got a clue.' *(PN25)

### vi) Developing (yet) another role

For the most part, practice nurses suggested that management of CFS/ME patients was best placed in primary care:

*'In primary care we look more at the whole patient rather than just one thing.' *(PN23)

*'I think to come to your normal surgery and be able to access that type of care would be very positive for patients.' *(PN28)

They suggested that management should involve a dual approach – to identify or exclude any physical problems and to work psychologically with patients.

*'I think a sympathetic GP is definitely of primary importance and then erm assess any underlying problems making sure they are physically, er, any other physical abnormalities or problems are excluded erm and counselling, appropriate counselling, with a counsellor who understands the illness and knows about it.' *(PN24)

Most practice nurses expressed an interest in developing their role in the management of patients with CFS/ME, however a minority did not. This was principally amongst those who did not think CFS/ME was a disease and therefore that this was not a legitimate activity for the practice nurse:

*'I think the money could be better spent. I do think this is the modern disease and we are spending a lot of money on it and yet there's people who can't get their hips done but we are spending more and more money on counselling services.' *(PN9)

Moreover some practice nurses implied that they shouldn't be managing what was seen as essentially a mental health problem:

*'I wouldn't see myself as being specialist in it. Er I'm not psychiatric nurse trained, erm, I haven't got really that much interest in that. I'll still stick with all my acute *[patients].*' *(PN25)

*'I don't think we should be taking on psychological problems...I'd be like – I'd be looking at the clock thinking, you know, we've all got problems...get over it. And that sounds really harsh.' *(PN26)

Even respondents who did see the relevance of working with this patient group, identified a range of barriers that currently exist to expanding their role and particular training needs. The most apparent was identifying tensions with their current work-load:

*'I don't know how that is going to fit in when nursing and GP hours in primary care are stretched as they are 'cos more and more patients are accessing them and expecting more services within primary care.' *(PN3)

*'I'll just shove a broom up my bum and do that at the same time *[laughter]. *Seriously, if you look at what practice nurses are doing now, if, you know, someone suggested, er, well, yer, you know take that on as well.' *(PN25)

Some remarked on the insufficient numbers of patients with CFS/ME within their registered practice population which would mean they wouldn't develop and maintain sufficient expertise:

*'You learn by experience. Its all very well giving us a few training sessions but I don't think in the long run we see enough CFS to really know what we are doing.' *(PN1)

*'I think it's a very specialist and specialised area and I think for the amount of training that one would need to do and for the amounts of times that one is likely to see the patients I'm not sure that that is the best way to spend the money... I think its unrealistic...money, time...how many patients are we going to get in I wonder?' *(P21)

Several participants perceived financial barriers to any extension of their role; unless practices would financially benefit from this work, practice nurses felt that their GP employers would object to them developing a role in the management of patients with CFS/ME:

*'It's like anything; if the money's not behind it, it's not gonna get done'*. (PN23)

However, nurses did feel that their being involved in managing the condition could potentially be cost-effective:

*'A doctor is a very expensive resource...once you've got *[a diagnosis]*then you have to look at appropriately someone else appropriately managing them and I don't think that would necessarily have to be a G.P.' *(PN25)

A further barrier to working with CFS/ME patients was job satisfaction. Some participants described this coming from seeing patients' condition improve. Where participants considered CFS/ME to have a poor prognosis, it was felt there was little intrinsic reward in managing these patients and hence not a good use of their skills:

*'Most doctors, and I would say quite a few nurses as well, want to make people better, erm, and so there's always that you know that you're failing, you're not making them feel better, you now, you like to say "oh yes this is what you have, take this and it will all go away"...any clinician wants to make people better...you want to be able to see the actual difference in someone, erm, and I think with CFS that's not there and it may ultimately be there but it would take a long time.' *(PN28)

However, this contrasted with others who saw a therapeutic value in their role which could be satisfying:

*'I think if you had, you know the, if you knew exactly what was out there and what, you know, and had a, a network of, of different options open to you I think you know, I think you could actually feel you could do something, to actively help and support them that would be good.' *(PN13)

Nurses identified a number of ways which would facilitate the development of their role in this way. Since all had disclosed that their knowledge about CFS/ME was limited, they felt that training was needed that covered basic knowledge such as symptoms and presentation and diagnosis. Moreover practice nurses wanted an explicit protocol to follow:

*'Need to know more about ME ...what patients want, how to treat it, what the protocol is and what happens when patients don't fall in with the protocol. Where you can access information...and where you can refer them on.' (*PN3)

Some believed this could make management relatively straightforward:

*'really I think that once a diagnosis has been made and the support framework has been set up then I think it is a case of just following the guidelines really.' *(PN5)

In addition to knowledge and guidelines, respondents also wanted further training in psychological management, feeling unhappy about using psychological approaches for fear of causing harm:

*'You can't have anyone give psychological support to people, ah, because it's quite a dangerous area really.' *(PN23)

Participants believed their training needs were substantial and were sceptical about whether these would be adequately met by within current arrangements:

*'It's like everything else they'd give us a two-day course and expect us to do it...it's silly, it's like giving you a two day course in diabetes and say "go and see the diabetic patients".' *(PN26)

Practice nurses also indicated that even if they were to undergo adequate training about CFS/ME and take a greater role in management, they would still require support, both during the training process and subsequent supervision, to support them in their work:

*'I'd want somebody who, really knew what they were talking about to be there for support and information for me. So that if...I'm stuck I can go to somebody that really does know a lot about it. Even after I've had the training and know a bit about it I'd want somebody there, you know a lot about it that I could, get support from, and questions answered and stuff like that.' *(PN12)

## Discussion

### Summary of main findings

Although practice nurses were aware of CFS/ME as a clinical condition, they had limited knowledge and experience of the clinical features of the illness, its aetiology and appropriate management strategies. They were aware that there is no definitive diagnostic test available making diagnosis difficult, and understood that CFS/ME is viewed as a contentious illness with the diagnosis itself held with some scepticism. Most practice nurses viewed CFS/ME in biopsychosocial terms, though others had less holistic or coherent illness models.

Practice nurses reported receiving little formal training about CFS/ME with the majority of their knowledge of CFS/ME being gained by personal research or experience, and they explained how encounters with people with CFS/ME outside of work generated acceptance, sympathy and interest in the condition; and encounters involving close personal friends particularly effective at forming strong opinions on the causative factors (and lack of blame) of CFS/ME.

Many nurses were keen to expand their current knowledge and gain more experience in the care of patients with CFS/ME, and felt that such a role embraced their present strengths of organization and motivation in chronic disease management, and the commonly held view that nurses are more approachable and sympathetic than other health care professionals and have skills in educational and motivational intervention. However, nurses were apprehensive about the prospect of taking on a new role without sufficient training and that they expressed doubts about whether their GP/FP employers' would be happy for them to expand their role in this area, given the demands of the Quality and Outcomes Framework of the GP Contract [23]. They recognised that their knowledge about CFS/ME was limited, and therefore any training which was provided would have to include basic knowledge about the condition and an explicit protocol and training in psychological interventions. They also described how they would need on-going support, both during the training process and when taking on the proposed role, through formal supervision, particularly from those with psychological expertise.

### Strengths and Limitations

This is the first study to report on the attitudes of practice nurses towards CFS/ME and their views highlight the potential barriers to the development of this role with this patient group. Data are presented from interviews with practice nurses over a wide geographical area and drawn from suburban, rural and inner city areas. This purposive sampling enabled us to access a range of views. Using authors from different professional and academic backgrounds is a recognised technique for increasing the trustworthiness of the analysis [21].

A limitation was the low response rate, with many invited nurses declining to participate stating that this was because they perceived they had a lack of knowledge about CFS/ME. The opinions expressed in the interviews may not be similar to all nurses in all practices, particularly those working in practices not participating in the FINE trial, who may be even less well informed than those who agreed to be interviewed. It is important to emphasize that practice staff, including nurses, in practices participating in the FINE trial [19], had little involvement in the actual study which simply involved referral to the trial by family physicians.

### Comparison with existing literature

CFS/ME is seen as a contentious illness by many of the practice nurses in this study. Some respondents viewed the diagnosis of CFS/ME with scepticism, similar to descriptions of the views of GPs/FPs in previous studies [11,12], where GPs/FPs did not consider CFS/ME to be a genuine illness, nor feel confident in neither its diagnosis nor management [3]. The issue is different for nurses who, unlike GPs/FPs, do not have the responsibility for making the diagnosis, but who still need to believe in the condition to manage the patient empathetically. Practice nurses described how the diagnosis of CFS/ME is not the end-point for the professional or the patient [24]. Nurses in this study gained information about CFS/ME from personal friends and their own experience which was powerful in influencing their views on the condition. This resonates with the sources of evidence that GPs/FPs report using to inform their knowledge base about CFS/ME [14].

The NICE guidelines suggest that healthcare professionals should aim to establish a supportive and collaborative relationship with patients with CFS/ME to facilitate their effective management [6]; other guidelines stress that where possible CFS/ME should be managed locally [4], and practice nurses interviewed concurred with these suggestions. The NICE guidelines allude to a role for practice nurses as care coordinators and suggest that graded exercise and CBT (Cognitive Behavioural Therapy) approaches should be used in the management of patients with CFS/ME.

Respondents suggest that the holistic approach attributed to nurses [17] would be effective in the management of patients with CFS/ME; they were unclear, however, about what the current evidence-base for management of CFS/ME is. Whilst they could describe a range of psychological approaches they already used in their current role, they tended to be limited to supportive listening and counselling, and they perceived that more formal training in psychological approaches would be necessary for working with patients with CFS/ME. This is consistent with previous findings [18] that demonstrated that practice nurses lack confidence in managing psychological problems in patients with mental health problems. This is not, however, peculiar to nurses: GPs/FPs also often lack confidence in taking a psychological approach with patients with CFS/ME [25] and commonly devalue their skills in psychological management of patients with unexplained symptoms [26].

### Implications for future research and clinical practice

If nurse-led therapeutic interventions are to be implemented in clinical practice, the training needs identified within this paper must be addressed. Practice nurses identified these to be across the entire spectrum including the requirement for knowledge about the epidemiology, aetiology, clinical features, diagnosis, and management of CFS/ME. CFS/ME as a condition needs to be demystified. In addition, they highlighted their own insecurities and lack of knowledge of the evidence-base about the different management approaches of patients with CFS/ME. Findings suggest that evidence-based diagnosis and management pathways would be vital for the effective delivery of any intervention in primary care for patients with CFS/ME and that nurses may have a role within a multidisciplinary approach. The relative rarity of CFS/ME in any one practice population (especially compared with, say, asthma or diabetes), would make it inefficient and difficult for practice nurses to develop and sustain expertise, and this is recognized by the practice nurses in this study. This is a critical issue for the management of CFS in primary care which would suggest that a multidisciplinary service based in a PCT (Primary Care Trust) rather than in individual practices may be more appropriate. The practice nurse role would then be limited to identification of patients with CFS/ME and referral to such a team, and offering support to patients (a role suggested by some nurse in this study) rather than developing skills in the active management of such patients.

The scepticism displayed by some of the practice nurses interviewed, reflecting that seen in other members of the medical profession, must be challenged when producing future training strategies. If practice nurses are to have a role in the management of patients with CFS/ME in primary care, the attitudes of this group and their beliefs about CFS/ME and its management will need to be addressed. It is vital that negative attitudes expressed by some respondents in this study are challenged as these attitudes and lack of knowledge will make it difficult to implement current policy and guidelines [6] in UK primary care. Education and training, at least initially incorporating these beliefs but later challenging them, will be needed.

Practice nurses also expressed the need for protocols to support them in the management of CFS/ME and they could usefully be directed to the emerging "Map of Medicine" guidelines [27]. Knowledge acquisition around the current evidence-base for treatment alone will be insufficient. Practice nurses need training to develop appropriate skills such as the competent use of a CBT approach which is evidence-based for patients for CFS/ME, unlike the counselling approach which practice nurses in this study suggested might be the appropriate intervention. In addition, the nurses in our study expressed a need for supervision if they are to take on a role in the management of patients with CFS/ME. This has implications for the cost of providing such a service either at practice or PCT level.

## Conclusion

Practice Nurses in this study demonstrate limited understanding of the evidence-base for treatment of CFS/ME, particularly psychological therapies, describing management options in terms of advice giving, self-help or counselling. Practice Nurses largely welcomed the potential development of their role in this area, but identified barriers and training needs which must be addressed to enable them to feel confident managing of patients with this condition. Training must begin by addressing negative attitudes to patients with CFS/ME.

## Competing interests

The authors declare that they have no competing interests.

## Authors' contributions

CCG designed and managed this qualitative study. She contributed to the data analysis and drafted the paper. She is guarantor for the study and paper. RD, JS and NS contributed to the design of the study and interview schedules, collected and analysed data, and contributed to the writing the paper. KL contributed to data analysis and revising the paper. SP contributed to the data analysis and writing and revising the paper. All authors read and approved the final manuscript.

## Pre-publication history

The pre-publication history for this paper can be accessed here:


